# Potential plasma biomarkers for progression of knee osteoarthritis using glycoproteomic analysis coupled with a 2D-LC-MALDI system

**DOI:** 10.1186/1477-5956-10-36

**Published:** 2012-06-06

**Authors:** Isao Fukuda, Takeshi Ishihara, Shigeki Ohmachi, Ikue Sakikawa, Atsushi Morita, Minoru Ikeda, Shoji Yamane, Tomoko Toyosaki-Maeda, Yoshihiko Takinami, Hiroyuki Okamoto, Yoshito Numata, Naoshi Fukui

**Affiliations:** 1 Shionogi Pharmaceutical Research Center, Shionogi & Co. Ltd, Toyonaka-Shi, Osaka, 561-0825, Japan; 2 Shionogi Innovation Center for Drug Discovery, Shionogi & Co. Ltd, Kita-ku, Sapporo, 001-0021, Japan; 3 Department of Pathomechanisms, Clinical Research Center, National Hospital Organization Sagamihara Hospital, Sagamihara, Kanagawa, 228-8522, Japan

**Keywords:** Osteoarthritis, 2D-LC-MALDI, Biomarker, Glycoprotein, Proteomics

## Abstract

**Background:**

Although osteoarthritis (OA) is a highly prevalent joint disease, to date, no reliable biomarkers have been found for the disease. In this study, we attempted to identify factors the amounts of which significantly change in association with the progression of knee OA.

**Methods:**

A total of 68 subjects with primary knee OA were enrolled in the study. These subjects were followed up over an 18-month period, and plasma and serum samples were obtained together with knee radiographs every 6 months, i.e., 0, 6, 12 and 18 months after the enrollment. Progressors and non-progressors were determined from the changes on radiographs, and plasma samples from those subjects were subjected to *N*-glycoproteomic 2D-LC-MALDI analysis. MS peaks were identified, and intensities for respective peaks were compared between the progressors and non-progressors to find the peak intensities of which differed significantly between the two groups of subjects. Proteins represented by the chosen peaks were identified by MS/MS analysis. Expression of the identified proteins was evaluated in synovial tissues from 10 OA knee joints by *in situ* hybridization, western blotting analysis and ELISA.

**Results:**

Among the subjects involved in the study, 3 subjects were determined to be progressors, and 6 plasma and serum samples from these subjects were subjected to the analysis together with another 6 samples from the non-progressors. More than 3000 MS peaks were identified by *N*-glycoproteomic 2D-LC-MALDI analysis. Among them, 4 peaks were found to have significantly different peak intensities between the progressors and non-progressors. MS/MS analysis revealed that these peaks represented clusterin, hemopexin, alpha-1 acid glycoprotein-2, and macrophage stimulating protein, respectively. The expression of these genes in OA synovium was confirmed by *in situ* hybridization, and for clusterin and hemopexin, by western blotting analysis and ELISA as well.

**Conclusions:**

In this study, 4 potential biomarkers were identified as potential prognostic markers for knee OA through *N*-glycoproteomic analysis. To the best of our knowledge, this is the first report for the use of glycoproteomic technology in exploring potential biomarkers for knee OA.

## Background

Osteoarthritis (OA) is the most prevalent joint disease in industrialized countries [1]. As it is an age-related disease, the number of patients suffering from OA is increasing dramatically in aging societies [2]. Although OA can affect any synovial joint, OA of the knee joints is of particular concern because of its prevalence and close association with disability [3,4].

Knee OA is a highly heterogeneous disease in terms of progression. Previous studies have indicated that the course of progression of knee OA differs considerably among patients and knee joints; some OA knees may undergo rapid progression, while others remain almost unchanged for years or decades [5-7]. To establish the optimum treatment protocol and to facilitate drug development, it is important to identify patients at risk of disease progression. Investigators have attempted to establish reliable biomarkers that can predict the progression of the disease [8]. However, despite these efforts, no surrogate protein biomarkers have been used clinically, primarily due to the lack of predictability. Therefore, it is necessary to establish reliable biomarkers for OA.

Proteomic analysis coupled with mass spectrometry (MS) is a powerful tool for exploring biomarkers, and this approach has also been used in the discovery of OA biomarkers [9]. Attempts to discover biomarkers in plasma are often hampered by the paucity of target proteins, because plasma contains a large variety of proteins at various concentrations ranging over ten orders of magnitude [10]. Various techniques have been developed to overcome this problem. Among them, depletion of highly abundant proteins by affinity chromatography has been demonstrated to be highly effective for biomarker discovery [11]. Fractionation of plasma proteins by multi-dimensional liquid chromatography is also useful for finding low-abundant proteins [12]. Enrichment focused on posttranslational modifications (PTMs) is another powerful strategy to concentrate target proteins [13,14].

Glycosylation, one of the most common forms of PTM of proteins, plays important roles in the maintenance of protein stability, binding of ligands to receptors, and adhesion of cells to the matrix [15,16]. Thus, enrichment of glycosylated proteins should be an effective procedure for discovering biomarkers in blood. In fact, we have recently demonstrated that glycoproteomic analysis is a powerful tool for finding novel biomarkers in plasma [17]. This method may be used for the exploration for OA biomarkers, because matrix components of articular cartilage, such as proteoglycans, are known to be highly glycosylated [18]. However, the proteomic analyses focused on PTMs have a limitation that the result of the analysis reflects both the amount of total protein and the level of modification on that protein. In case of glycosylation modification, the analysis may be hampered by the complexity of modification, which stems from the heterogeneity of carbohydrate structures.

In the present study, a glycoproteomic approach using 2D-LC-MALDI was used to discover factors the concentration of which changes with the progression of knee OA. Recently, we conducted a follow-up study of knee OA patients, and confirmed that the course of radiographic progression of knee OA differed considerably among patients [19]. Using plasma obtained from these subjects, we compared the samples from the subjects who were in the middle of disease progression with those from the subjects who were in a stable condition of the disease. Four proteins were found to fulfill our criteria, which we expect to serve as prognostic factors for the disease.

## Results

### Exploration of biomarker candidates by glycoproteomic analysis

As described earlier, the purpose of this study was to find factors the amounts of which increase or decrease in association with disease progression, expecting that such factors would serve prognostic biomarkers for knee OA. Thus, the factors we attempted to find were termed biomarker candidates hereafter. To find biomarker candidates, *N*-Glycoproteomic 2D-LC-MALDI analysis was carried out using approximately 30 μL of plasma samples. Prior to this study, we evaluated the performance of our proteomic analysis with human plasma samples, which demonstrated excellent sensitivity (down to 10^3^ pg/mL) and sufficient reproducibility (CV <  34%) of the system [17].

**Figure 1:**
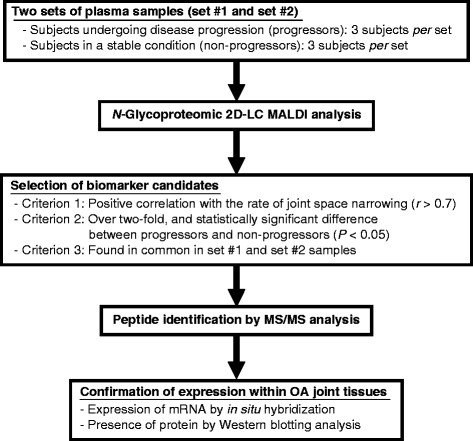
Schematic flowchart of analysis strategy.

Glycoproteomic analysis yielded 3456 and 3357 MS peaks in set #1 and set #2 samples, respectively, using DeView software. We chose the peaks for potential biomarkers by the following three selection criteria (Figure 1). First, for each MS peak, linear regression analysis was performed between the rate of narrowing of joint space width (JSW; this indicates the rate of disease progression) and the intensity of the peak, and the peak was taken as a candidate when the correlation coefficient (*r*) was above 0.7. By these criteria, 435 and 350 peaks were chosen for set #1 and set #2 samples, respectively. Next, the MS peaks were chosen with the condition that the average of the intensity for the progressors was over two-fold greater than that for the non-progressors, and that the difference in intensity was statistically significant between the progressors and the non-progressors (*P*  <  0.05 by Mann–Whitney *U* test). Through this selection, 176 and 92 MS peaks were chosen in set #1 and set #2 samples, respectively. Next, we chose peaks found in common in both set #1 and set #2 samples, and four MS peaks were finally selected for further analysis.

### Identification of biomarker candidates

The glycoproteins represented by the four selected MS peaks were subjected to MALDI-TOF/TOF MS analysis for identification (Table 1). Plasma samples collected from the progressors were used for the analysis, after confirming that the peaks had sufficient intensities for MS/MS analysis. The obtained MS/MS data were analyzed using Mascot software, and the reliability of identification was confirmed by the significant high values of the scores. The data were also analyzed by the MS-Tag software (ProteinProspector v 5.10.1; http://prospector.ucsf.edu/prospector/mshome.htm), and identical results were obtained. In the search using the Mascot software, the possibility was considered a possibility of conversion of asparagine to aspartic acid by deglycosylation with PNGase F, which causes a mass increase of 0.98 Da. To confirm the identification of the protein, the analysis was repeated at least twice for each MS peak. After identification, the presence of the consensus Asn-X-Thr/Ser motif of *N*-linked glycopeptides was confirmed with each determined sequence (Table 1).

**Table 1:** Biomarker candidates identified by proteomic analysis

**Peak cluster (sample set)**	Correlation coefficient (*r*)*	Fold change**	*P *value***	**Identified peptide**	**Protein name**	**Accession No.**	**Mascot score**	**Error, Da (ppm)**
2932 (#1)	0.799	6.4	0.012	LVPVPIT**NAT**LDRITGK	α-1 acid glycoprotein-2	NP_000599	98	0.131 (72.4)
2424 (#2)	0.769	2.9	0.007					
4512 (#1)	0.894	2.3	0.199	ML**NTS**SLLEQLNEQFNWVSR	Clusterin	NP_976084	66	0.052 (21.6)
3819 (#2)	0.812	3.5	0.009					
4878 (#1)	0.916	3.1	0.028	GTG**NDT**VLNVALLNVISNQECNIK	Macrophage stimulating protein	NP_066278	43	0.221 (85.3)
4126 (#2)	0.720	2.1	0.061					
4483 (#1)	0.731	19.3	0.005	GHGHR**NGT**GHG**NST**HHGPEYMR	Hemopexin	NP_000604	96	0.118 (49.0)
3797 (#2)	0.916	10.0	0.007					

*N*-Glycosylation motifs (N-X-S/T) are indicated in bold, and glycosylated asparagine residues are underlined.

*Correlation coefficient between the peak intensity and the rate of JSW narrowing. **Calculated from the mean intensity of progressors (n = 3) to that of non-progressors from respective sample set (#1, #2). ***Calculated by Student’s *t*-test.

Among the four identified proteins, a peptide fragment of G^235^HGHRNGTGHGNSTHHGPEYMR^256^ (*m/z* 2,414.1 with two glycosylated asparagine residues and an oxidized methionine residue) was found to have originated from hemopexin. Two *N*-glycosylation motifs were found within this fragment. For this fragment, the peak intensities of progressors were 19.3-fold and 10.0-fold greater than those of non-progressors in the set #1 and set #2 samples, respectively. This difference was greatest among the four biomarker candidates found in this study, suggesting that hemopexin may be a promising biomarker for the disease.

A peptide fragment of L^26^VPVPITNATLDRITGK^42^ (*m/z* 1,809.1 with one glycosylated asparagine residue) was identified to be a peptide generated from alpha-1 acid glycoprotein-2 (AGP-2). The N-terminal amino acid sequence of the mature AGP-2 molecule was Q^19^IPLCANLVPVPITNATLDRITGK… according to the UniProt/Swiss-Prot database (version 117). Thus, the identified AGP-2 fragment lacked the first seven amino acids at the N-terminus. Although the samples underwent trypsin digestion prior to the analysis, this treatment was unlikely to cause the loss of the seven amino acids, because trypsin cleaves proteins only at the C-terminus of arginine (R) or lysine (K). Therefore, the identified AGP-2 fragment was considered to be generated *in vivo* by the processing of an endogenous protease. This fragment was identified with a sufficiently high score by Mascot search, and was observed consistently in all plasma samples analyzed in this work. Moreover, the samples from the progressors and non-progressors were completely discriminated by the MS peak intensities for this fragment. Thus, this cleaved form of AGP-2 could be a novel but reliable prognostic factor for the disease.

**Figure 2:**
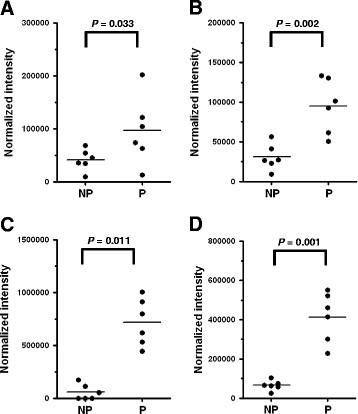
**Comparison of MS peak intensities of potential biomarkers between progressors and non-progressors.** Results are shown for clusterin (**A**), MSP (**B**), hemopexin (**C**), and AGP-2 (**D**). P and NP represent progressors and non-progressors, respectively. *P*-values were determined by Mann–Whitney *U* test.

To evaluate the usefulness as a biomarker, MS peak intensities of the plasma samples were compared between the progressors and non-progressors. As anticipated, the peak intensities with the progressors were significantly greater than those with the non-progressors for all four potential biomarkers (Figure 2). The statistical significance of the difference was the least with clusterin (*P*  =  0.033), followed by hemopexin and macrophage stimulating protein (MSP), and the greatest with AGP-2 (*P*  =  0.001). For hemopexin (Figure 2C) and AGP-2 (Figure 2D), progressors and non-progressors were completely separated by the MS peak intensities.

### Serum levels of potential biomarkers

Among the four proteins identified, the concentrations of clusterin and hemopexin in the serum samples were determined by ELISA. The results of this assay indicated that the concentration of neither protein differed significantly between the progressors and non-progressors (Table 2).

**Table 2:** Concentrations of clusterin and hemopexin in serum samples

** Protein**	**Concentration (μg /mL)**		Statistical significance (*P*)*
	**Progressors (*n* = 6)**	**Non-progressors (*n* = 6)**	(*P*)*
Clusterin	239 ± 18.5	243 ± 18.4	N.S. (0.353)
Hemopexin	1,241 ± 133	1,259 ± 36.3	N.S. (0.373)

*Statistical analyses were performed by Mann–Whitney *U* test. N.S., not significant.

### Expression of biomarker candidates in OA synovial tissue

**Figure 3:**
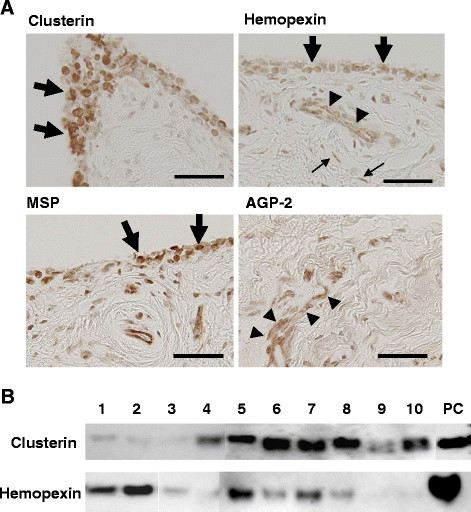
**Expression of candidate biomarkers in synovial tissues collected from OA knee joints.** (**A**) Results of *in situ* hybridization for clusterin, hemopexin, MSP, and AGP-2 mRNA in OA synovial tissue. Clusterin, hemopexin, and MSP expression were observed primarily in synovial lining cells (thick arrows), while the expression of AGP-2 was mostly observed in endothelial cells (arrowheads). Expression of hemopexin was also observed in endothelial cells (arrowheads) and fibroblast-like cells (thin arrows). Bars, 50 μm. (**B**) Results of western blotting analysis of synovial tissue lysates. Clusterin and hemopexin were confirmed in all samples. Numbers above the lanes indicate numbers of synovium samples. PC: positive control.

We next investigated the expression of the four biomarker candidates within OA synovial tissue. For this, synovial tissues were obtained from 10 end-stage OA knees at the time of prosthetic surgery. First, mRNA expression for these molecules was investigated by *in situ* hybridization, which confirmed abundant expression of all 4 genes coding the candidate proteins in the synovial tissues from OA knees (Figure 3A).

For clusterin and hemopexin, their presence in the synovial tissues was examined by western blotting analysis using specific antibodies for respective proteins. The results of this analysis showed that these proteins were in fact present at substantial levels in the synovial tissues obtained from OA-affected knee joints (Figure 3B).

## Discussion

To date, several proteins are known to associate with the progression of knee OA. Cartilage oligomeric matrix protein (COMP) in serum [6,20] and the C-telopeptide of type II collagen (CTX-II) in urine [21-23] are the examples. Hyaluronic acid and C-reactive protein (CRP) in serum may also be associated with the progression of the disease [24-27]. However, none of these markers are reliable enough to be used clinically. This lack of established biomarkers for OA may be partly ascribed to the difficulty in specifying the phase of disease progression. In most previous studies, blood or urine samples were obtained only once, at the beginning of the study period. Such samples may not be necessarily obtained during the progression of the disease, because the progression may have started after the collection of the initial samples. Considering this possibility, in the present study, we obtained knee radiographs repeatedly during the study period, and specified the samples acquired in the middle of disease progression. Due to this study design, we consider the four molecules identified here would be highly promising as prognostic biomarkers for the disease.

Another advantage of this study is the application of a glycoproteomic approach to the discovery of biomarkers. To our knowledge, this is the first study to use this technology for the exploration of biomarkers for OA. In this approach, glycoproteins in plasma were specifically concentrated using Affi-Gel Hz hydrazide gel, and the trypsin-digested glycoproteins were subjected to comprehensive analysis by 2D-LC-MALDI. In understanding the result, it should be noted that the changes in peak intensities reflect not only the change in the amount of a protein but also the change in the level of glycosylation on that protein. Considering this issue, for hemopexin and clusterin, we determined the amounts of the proteins by Elisa. These measurements revealed that the concentration of neither protein differed significantly between the progressors and the non-progressors. Considering this result together with the differences in the amounts of glycosylated proteins, we currently assume that for these proteins, the level of glycosylation may increase during the progression of the disease. In order to validate this assumption, we have performed western blotting analysis, but failed to demonstrate the anticipated changes in glycosylation levels between the progressors and non-progressors, possibly due to technical limitations of the method (Additional file 1). Recent studies have shown that the changes in the patterns of glycosylation on hemopexin and clusterin may be useful for the diagnosis for hepatocellular carcinoma [28,29]. We consider similar scenarios might be true with these two proteins for knee OA patients.

For the remaining AGP-2 and MSP, we are currently unable to determine the protein concentrations, and it is therefore still not clear whether the observed differences in peak intensities between the progressors and non-progressors were related to the difference in the amounts of proteins and/or in the levels of glycosylation. Further studies are necessary to clarify why the peak intensities for these proteins differed between the two groups of subjects.

Among the four identified biomarkers, clusterin is a heterodimeric glycoprotein which is known to be expressed in a variety of biological events, such as inflammation, tissue injury, cold stress, and oxidative stress [30,31]. In these conditions, clusterin is expressed by various types of cells, and exhibits protective effects on the cells. In OA, both chondrocytes and synoviosytes are known to express clusterin [32-36]. As mentioned above, we assume that the level of glycosylation on clusterin would elevate in those cells in association with the progression of the disease.

Hemopexin is one of the acute-phase response proteins produced primarily by hepatocytes [37]. This protein is abundant in plasma, and plays significant roles in inflammation and tissue repair, likely by suppressing the adhesion of polymorphonuclear leukocytes [38]. During tissue repair, hemopexin suppresses the release of proinflammatory cytokines from macrophages [39]. In analogy to clusterin, we observed that the level of glycosylation on hemopexin, but not the amount of total protein, was increased in patients undergoing disease progression. We assume that the increased glycosylation on this protein would reduce the anti-inflammatory effects of hemopexin, which could facilitate disease progression. This possibility may be worth examined by future studies.

AGP-2, also known as orosomucoid-2, is another acute-phase response protein that exists abundantly in plasma. AGP-2 is secreted by hepatocytes and released into the circulation in highly *N*-glycosylated forms [40]. The concentration of AGP-2 in plasma is known to elevate with tissue injury, inflammation, and certain types of cancer [41]. In interpreting the present findings, it should be noted that the peptide identified by MS/MS was not the entire AGP-2 molecule but a processed fragment lacking an N-terminal region. Although speculative, it seems plausible that the amount of this fragment increased in the progressors by the increase in protein processing, rather than by the rise in the glycosylation level. This suggests the possibility of a specific enzyme being induced in OA knees during the progression of the disease, which could yield this AGP-2 fragment. Such processing may be involved in the pathology of OA through the modulation of the biological activity of AGP-2.

Similar to hemopexin and AGP-2, MSP is synthesized primarily by hepatocytes as an inactive precursor and released into the circulation [42]. In the blood, most MSP is present in this biologically inactive form. To become biological active, the precursor should be processed into MSP-α and MSP-β, and these two proteins should be dimerized by disulfide bonds. This active MSP (α-β heterodimer) is known to have biphasic effects on macrophages. While the protein enhances spreading, migration and phagocytosis of macrophages, it may also inhibit macrophage production of inflammatory mediators such as TNF-α [43]. Thus, increased levels of MSP in OA patients may account for the unique pathology of OA synovium, which is characterized by macrophage infiltration without overt signs of inflammation. In this study, we found that the peak intensity for MSP-β was increased in association with disease progression. Since we were unable to determine whether this increase was caused by the increase in the amount of MSP-β protein or by the enhancement of glycosylation on this protein, the significance of the change in peak intensity is not yet clear. Further studies are necessary to determine its significance in the pathology of OA.

Thus, we have reported that the concentrations of four molecules are increased in plasma of knee OA patients in association with the progression of the disease, suggesting that these molecules have a potential to be prognostic markers for the disease. Hopefully, future studies demonstrate reliability of these factors as biomarkers.

## Conclusions

To establish a prognostic factor for knee OA is indeed a challenging task. In this work, we employed state-of-the-art proteomic technologies, and attempted to identify factors the amounts of which change in association with the progression of the disease. Four factors were found in the patients’ plasma whose concentrations increased significantly during disease progression, and those factors are expected to serve as prognostic factors for the disease. The result of this study demonstrated that *N*-glycoproteomic analysis could be a powerful tool to discover biomarker candidates that exits at very low concentrations in samples.

## Methods

### Study subjects and collection of plasma samples

This study was approved by the institutional review boards of the participating institutions, and informed consent was obtained in writing from each subject. To obtain plasma samples from the subjects with and without progression of knee OA (progressors and non-progressors, respectively), we followed up knee OA patients over an 18-month period, which was conducted as a part of a 3-year follow-up study of knee OA patients [19]. The subjects had been diagnosed to have primary knee OA with radiographic manifestations. Subjects with any serious health problems other than knee OA were excluded from the study.

In the 18-month study period, plasma and serum samples were obtained every 6 months, together with knee radiographs. To prepare the plasma samples, peripheral blood was drawn into EDTA-containing tubes, and the plasma was separated by centrifugation. For serum samples, blood was obtained in collection tubes, and the serum was obtained by centrifugation after 60 min of incubation at room temperature. The plasma and serum samples were stored at −80°C until use.

The progression of knee OA was determined by the changes in JSW between the femur and tibia in the involved compartment of the knee, which was measured on postero-anterior radiographs obtained in a weight-bearing position [44,45]. In this study, progressors and non-progressors were determined using the following conditions. Progressors were patient in whom the JSW decreased continuously for 18 months or three consecutive 6-month periods (four consecutive follow-up visits) in at least one knee. In non-progressors, the JSW did not show any detectable changes for three consecutive 6-month periods in either knee. For each progressor, the annual rate of JSW narrowing was determined for every 6-month period. If a progressor showed disease progression bilaterally, the higher rate was chosen for the subject.

### Selection of plasma samples for 2D-LC-MALDI analysis

Among all subjects enrolled in the study, 3 were determined to be progressors, and another 12 were categorized as non-progressors according to our criteria. The rate of JSW narrowing for these 3 progressors was 0.71 – 4.4 mm/year. Among the 12 non-progressors, 3 patients were chosen for the study considering equivalence to the progressors in age, BMI, and radiographic severity of OA [46] (Table 3). The samples from these 3 non-progressors were analyzed together with those from the 3 progressors.

**Table 3:** Demographic and clinical characteristics of patients who provided plasma and serum samples for analysis

**Age**	**Sex**	**Height (cm)**	**Body weight (kg)**	**BMI**	K/L grade*	
					**Right**	**Left**
Progressors						
71	Female	150	58	25.8	Grade 2	Grade 3
76	Female	152	65	28.1	Grade 2	Grade 2
67	Female	157	57	23.1	Grade 1	Grade 2
Non-progressors						
75	Male	165	70	25.7	Grade 1	Grade 2
71	Female	151	70	30.7	Grade 2	Grade 3
72	Female	145	57	27.1	Grade 2	Grade 2

*Kellgren–Lawrence grade [46] on enrollment.

During the 18-month follow-up period, these 6 patients (3 progressors and 3 non-progressors) visited the clinic 4 times as requested, and thus 4 plasma samples were obtained from each subject at 6-month intervals. Among these 4 samples, those obtained at the second (set #1) and third visits (set #2) were used for the analysis. These samples were chosen because at those visits, it could be determined exactly whether a subject was undergoing disease progression or was in a stable (non-progressing) condition, because the changes in JSW were known for both the 6-month period before the visit and the next 6-month period after the visit. If JSW reduced successively in these two 6-month periods, the subject was surely in the middle of disease progression at that visit.

### 2D-LC-MALDI analysis focusing on *N*-glycoproteins

In this study, all plasma samples obtained from the patients were analyzed independently, and no samples were pooled or mixed for the analysis. The method for the analysis of *N-*glycoproteins in plasma samples was described previously [17]. Briefly, to deplete highly abundant proteins, 100 μL of plasma sample was passed through MARS-human 7 LC columns (4.6  ×  100 mm; Agilent Technologies, Santa Clara, CA). *N*-glycoproteins in the flow-through were concentrated using an Affi-Gel Hz Immunoaffinity Kit (Bio-Rad Laboratories, Hercules, CA) and digested with trypsin. Then the digested *N-*glycopeptides were recovered using PNGase F (New England Biolabs, Ipswich, MA), and aliquots of 10 μg were separated by 2D-LC (Ultimate 3000; Dionex, San Francisco, CA) onto Prespotted AnchorChip target plates (Bruker Daltonics, Bremen, Germany). All MS and MS/MS measurements were performed using an Ultraflex II MALDI TOF/TOF mass spectrometer (Bruker Daltonics). Peak detection parameters were set as follows: mass range, *m/z* 800 – 5,000; cutoff signal intensity, 1,000; laser iterations, 1,000 shots; mass tolerance, 100 ppm for precursor ion and 0.8 Da for fragment ion. The obtained data were analyzed using the Mascot search engine (Matrix Science, London, UK) against the International Protein Index (IPI) database with variable modifications as follows: cysteine carbamidomethylation, methionine oxidation, and PNGase F-catalyzed conversion of asparagine to aspartic acid.

Quantitative analysis of the MS data set was performed with DeView software (MCBI, Tsukuba, Japan). Each peak intensity of identical *m/z* was normalized and clustered using the following parameters: mass range, *m/z* 800 – 5000; cutoff signal intensity, 1000; mass tolerance for clustering identical peaks, ± 0.4 Da; fraction number for clustering identical peaks, within 8. After integrating each peak, the total peak list (*m/z vs* clustered peak intensity) was generated for statistical analysis.

### ELISA

Serum levels of clusterin and hemopexin were determined by human clusterin Quantikine ELISA (R&D Systems, Minneapolis, MN) and human hemopexin ELISA (Kamiya Biomedical Co., Seattle, WA), respectively, in accordance with the manufacturers’ instructions. The assays were performed in duplicate.

### Acquisition of synovial tissues

To evaluate the expression of possible prognostic factors in the synovium, synovial tissues were obtained from 10 end-stage OA knees at the time of prosthetic surgery. Immediately upon harvest, the synovial tissue was divided equally into two parts each weighing 200 – 400 mg wet weight. One portion was snap-frozen in liquid nitrogen and stored at −80°C for western blotting analysis. The other was processed for *in situ* hybridization.

### Western blotting analysis

Tissue lysates of OA synovium were prepared as follows. A sample of synovial tissue was pulverized while frozen in a tissue homogenizer (SK-200; Tokken, Chiba, Japan), and sample extraction buffer (8 M urea, 4% CHAPS, 65 mM dithioerythritol) containing protease inhibitors (Complete Protease Inhibitor Cocktail; Roche Diagnostics, Basel, Switzerland) was added at a rate of 10 mL per 1 g wet weight of the tissue. After gentle rotation at 4°C overnight, the sample was centrifuged at 15,000  ×  *g* for 15 min, and the tissue lysate was obtained as a supernatant. After determining protein concentration, the lysate was stored at −80°C until use.

The lysate containing 1 μg of protein was electrophoresed in each lane of a 4% – 12% SDS-polyacrylamide gel. The separated proteins were transferred onto a PVDF membrane, which was blocked with 5% skimmed milk in TBS for 1 h at 25°C. The membrane was then incubated with anti-human clusterin monoclonal antibody (1:1000, R&D Systems) or anti-human hemopexin polyclonal antibody (1:2500; Lifespan Biosciences, Seattle, WA) in TBST (0.05% Tween 20 in TBS) containing 5% skimmed milk overnight at 4°C. Subsequently, the membrane was washed 3 times with TBST for 5 min each time and then incubated with HRP-conjugated anti-mouse IgG polyclonal antibody (1:2000; GE Healthcare, Uppsala, Sweden) in TBST containing 0.5% skimmed milk for 1 h at 25°C. After washing 3 times with TBST for 5 min each time, the protein of interest was detected with ECL-plus (GE Healthcare) on a LAS-3000 image analysis system (Fuji Film, Tokyo, Japan).

### *In situ* hybridization

For *in situ* hybridization, synovial tissues were cut into small pieces (3-mm cubes) and fixed in 4% paraformaldehyde fixative at room temperature overnight. The tissues were then embedded in paraffin.

DIG-labeled cRNA probes for human clusterin and hemopexin were synthesized from PCR-generated cDNA fragment. The clusterin cDNA fragment was amplified using the primer pair, 5'-TTGGAGGCATGATGAAGACT-3' (forward) and 5'-TTCAGGAACTCCTCAAGCTG-3' (reverse). The hemopexin cDNA fragment was amplified using the primer pair, 5'-CCATTGCTCATCAGTGGCCC-3' (forward) and 5'-GTGAGTGCAGCCCAGGAGAC-3' (reverse). The MSP cDNA fragment was amplified using the primer pair, 5'-CGCACAAGCCGCAGTTCACG-3' (forward) and 5'-CCATCTTGGCTACTGGGACC-3' (reverse). The AGP-2 cDNA fragment was amplified using the primer pair, 5'-TTGCTGGGCTCCAAGTGACC-3' (forward) and 5'-CATCAAGGTCTTGGTGTCCC-3' (reverse). Each fragment was purified and subcloned into pCRII-TOPO vector (Invitrogen, Carlsbad, CA). The subcloned samples were linearized with the restriction enzyme *Hin*dIII as the templates of probes, and the labeled cRNA probes were synthesized with T7 RNA polymerase in the presence of digoxigenin (DIG)-labeled UTP (Roche Diagnostics) for antisense probe, or they were linearized with the restriction enzyme *Eco*RV, and the labeled cRNA probes were synthesized with Sp6 RNA polymerase for the sense probe. DIG incorporation of each probe was assessed by dot blotting. Paraffin sections (10 μm thick) were dewaxed in xylene and rehydrated through a graded alcohol series. The sections were treated with 3 μg/mL proteinase K (Roche Diagnostics) for 10 min at 37°C and acetylated with 0.25% acetic anhydride in 0.1 mol/L triethanolamine (pH 8.0) for 10 min. The sections were prehybridized at 52°C for 2 h with hybridization buffer containing 50% deionized formamide, 1× Denhardt’s solution, 15% dextran sulfate, 600 mM NaCl, 0.1% SDS, 5 mM EDTA, 0.25 mg/mL yeast tRNA, and 10 mM Tris–HCl (pH 8.0). Next, the slides were incubated in hybridization buffer containing 500 ng/mL DIG-labeled antisense or sense cRNA probe for 16 h at 55°C. After hybridization, the sections were rinsed with 5× sodium chloride-sodium citrate buffer (SSC) for 5 min at 50°C. The sections were washed twice with 2× SSC for 30 min at 50°C and thoroughly washed twice with 0.2× SSC for 30 min at 50°C. The sections were immersed in 5% normal rabbit serum for 30 min. The sections were then incubated with 0.4 μg/mL sheep anti-DIG antibody (Roche Diagnostics) for 60 min. The sections were incubated with biotinylated rabbit anti-sheep IgG (Vector Laboratories, Burlingame, CA), followed by treatment of avidin-biotin complex solution (Vector Laboratories). 3,3-Diaminobenzidine (DAB) was used as a chromogen. The sections were dehydrated with alcohol, cleared with xylene, coverslipped, and mounted with Entellan-new (Merck, Darmstadt, Germany). Photomicrographs of bright field images were obtained with an Olympus BH-2® microscope (Olympus, Tokyo, Japan).

## Abbreviations

OA: Osteoarthritis; PTM: Posttranslational modification; JSW: Joint space width; AGP: Alpha-1 acid glycoprotein; MSP: Macrophage stimulating protein.

## Competing interests

The expenses for this study were defrayed in part by Shionogi & Co. Ltd.

## Authors’ contributions

IF and TI designed and carried out the proteomic analysis and drafted the manuscript. SO and MI conceived and carried out the *in situ* hybridization studies and drafted the manuscript. IS and YT made substantial contributions to the data acquisition and interpretation in mass spectrometry analysis and western blotting analysis. AM helped in experimental design and in revising the manuscript. SY, T-MT, HO, and YN participated in study design and coordination. SY further helped in the selection of plasma samples used in this study. NF conceived the study, helped in experimental design, provided plasma samples, and critically revised the manuscript. All authors read and approved the final manuscript.

## Supplementary Material

Additional file 1 Western blotting analysis for detection of the changes of glycosylation forms in hemopexin and clusterin in the serum samples from OA patients. Click here for file
