# Supplementary material for: Potential plasma biomarkers for progression of knee osteoarthritis using glycoproteomic analysis coupled with a 2D-LC-MALDI system
Source: Proteome Sci. 2012 Jun 6;10:36. doi: 10.1186/1477-5956-10-36 (PMC3514375; doi:10.1186/1477-5956-10-36)
Supplement: Additional file 1 — Western blotting analysis for detection of the changes of glycosylation forms in hemopexin and clusterin in the serum samples from OA patients. [file 1477-5956-10-36-S1.doc]

**Additional file 1**

For hemopexin and clusterin, possible changes in glycosylation were investigated by western blotting analysis. The methods for this analysis are described below together with the result of the analysis.

**Methods**

The serum samples from 12 OA patients (6 progressors and 6 non-progressors), which had been used previously for 2D-LC-MALDI analysis, were used for the analysis. In this analysis, glycosylated proteins were condensed prior to the analysis by use of 3 types of lectins. Glycoproteins which bound to concanavalin A (ConA) and wheat germ agglutinin (WGA) were collected using commercially available kits containing lectin-conjugated resin (Glycoprotein Isolation Kits; Thermo Fisher Scientific, Waltham, MA), following the manufacturer’s recommended protocols. Glycoproteins which had the affinity to aleuria aurantia lectin (AAL) were obtained by AAL conjugated agarose (Vector Laboratories, Burlingame, CA). For this, 10 μL of serum sample was diluted to 600 μL with the binding/wash buffer (20 mM Tris-HCl, pH7.2, 150 mM NaCl, 0.1% Tween 20) containing complete protease inhibitor cocktail-EDTA free (Roche Diagnostics). The diluted serum was then incubated with the AAL agarose beads for 10 min at 25˚C. After appropriate washing, the agarose was recovered by centrifugation.

Western blotting analysis was performed using these concentrated glycoproteins and the original serum samples that had not undergone any treatment. The proteins that bound to lectin-conjugated resin or agarose were recovered in 200 μL of SDS sample buffer by incubation at 70˚C for 10 min. The protocol for the western blotting analysis is described in the Method section. In the analysis, clusterin and hemopexin were detected by mouse anti-human clusterin monoclonal antibody (1 μg/mL; R&D Systems), and mouse anti-human hemopexin monoclonal antibody (1 μg/mL; R&D Systems), respectively, as the primary antibodies. TrueBlot® ultra for anti-mouse Ig HRP-conjugated secondary antibody (1:10,000; eBioscience, San Diego, CA) was used to detect the primary antibodies that bound to the antigens. The presence of clusterin and hemopexin were finally detected with ECL-prime (GE Healthcare) on a LAS-3000 image analysis system (Fuji Film, Tokyo, Japan). Densitometric intensities for respective bands were quantified by Multi Gauge software (version 3.0, Fuji Film). Statistical analysis was performed using Mann-Whitney *U*-test.

**Results and Discussion**

In this western blotting analysis, hemopexin and clusterin were detected in all examined samples, irrespective of the difference in lectins used for protein concentration. Figure A shows representative results of this analysis. As shown in this figure, hemopexin or clusterin was detected as a single band in all samples, irrespective of the types of lectins used for pre-treatment, and no isoforms were detected for either protein.

Next, using the images obtained by the western blotting analysis, we attempted to detect changes of glycosylation levels between progressors and non-progressors. For this, the ratios of band densities were obtained between the lectin-treated samples and the untreated samples for respective samples, and these ratios were compared between progressors and non-progressors. The results of this comparison are shown in Figure B. As shown in this figure, statistical differences were not found with either protein between progressors and non-progressors, possibly due to a large individual difference and a limitation in quantitative capacity of the method.

Thus, we are currently unable to confirm the result of our 2D-LC MALDI analysis. This could partly be ascribed to the high analytic capacity of MALDI, but this also means that the results obtained by MALDI analysis may be difficult to confirm by other conventional methods.

**Figure A**

Figure A Representative results of western blotting analysis of samples from non-progressor (sample # 149) and progressor (sample #151). Serum samples were treated with three types of lectin-conjugated resin or agarose, and analyzed together with the untreated samples by western blotting analysis using primary antibody for indicated protein. ConA, concanavalin A; WGA, wheat germ agglutinin; AAL, aleuria aurantia lectin. For samples treated with AAL agarose, 10 (x 10) or 20 times (x 20) greater amounts of samples were loaded.

**Figure B**

Figure B Results of densitometric analysis. Densities of the bands detected by western blotting analysis shown in Figure A were quantified, and density ratio of the bands was obtained for each sample between the lectin-treated sample and untreated sample. Ratios are against untreated samples. ConA, concanavalin A; WGA, wheat germ agglutinin; AAL, aleuria aurantia lectin. Bars indicate mean values.
